# Blood borne Tau Aggregates and Their Role in Alzheimer's Disease Propagation

**DOI:** 10.1002/alz70855_106622

**Published:** 2025-12-25

**Authors:** Jesus Garcia‐Martin, Laura Vegas‐Gomez, Maria Angeles Arredondo‐Alcala, Antonia Gutierrez, Claudia Duran‐Aniotz, Ines Moreno‐Gonzalez

**Affiliations:** ^1^ Dept. Cell Biology, Faculty of Sciences, University of Malaga. IBIMA., Malaga, Spain; ^2^ Centro de Investigación Biomédica en Red (CIBER), Madrid, Spain; ^3^ Latin American Brain Health Institute (BrainLat), Universidad Adolfo Ibañez, Santiago, Chile; ^4^ The University of Texas Health Science Center at Houston, Houston, TX, USA

## Abstract

**Background:**

Alzheimer's disease (AD) is a neurodegenerative disorder characterized by the accumulation of extracellular amyloid‐β (Aβ) plaques and intracellular neurofibrillary tangles composed of hyperphosphorylated tau. While Aβ propagation via peripheral routes has been identified, the potential dissemination of tau aggregates beyond the central nervous system remains poorly understood. Evidence suggests that tau may spread in a prion‐like manner, with tau aggregates detected in peripheral tissues and biological fluids. However, the extent to which peripheral tau aggregates influence AD pathology is still unclear.

**Method:**

The objective of this study is to analyze whether peripheral tau aggregates contribute to disease progression by administering blood from tauopathy‐affected mice into P301S transgenic mice, a well‐established tauopathy model. Intravenous (i.v.) and intraperitoneal (i.p.) administration routes were used to evaluate systemic tau distribution, aggregation patterns, and potential central nervous system infiltration. The presence of tau aggregates in peripheral circulation and their interaction with brain pathology were assessed using behavioral, biochemical and histological analyses.

**Result:**

Our study indicates that the injection of blood borne tau aggregates via i.v. and i.p. is associated with an exacerbation of tau pathology in the brain, suggesting a potential interaction between peripheral and central tau dynamics.

**Conclusion:**

These results provide evidence that peripheral tau aggregates may act as pathological seeds, contributing to AD progression. Understanding the systemic dissemination of tau could offer novel insights into AD pathogenesis, identifying potential biomarkers and therapeutic targets for neurodegenerative diseases.

